# Need for Aeromedical Evacuation High-Level Containment Transport Guidelines

**DOI:** 10.3201/eid2505.181948

**Published:** 2019-05

**Authors:** Shawn G. Gibbs, Jocelyn J. Herstein, Aurora B. Le, Elizabeth L. Beam, Theodore J. Cieslak, James V. Lawler, Joshua L. Santarpia, Terry L. Stentz, Kelli R. Kopocis-Herstein, Chandran Achutan, Gary W. Carter, John J. Lowe

**Affiliations:** Indiana University School of Public Health, Bloomington, Indiana, USA (S.G. Gibbs, A.B. Le);; University of Nebraska Medical Center College of Public Health, Omaha, Nebraska, USA (J.J. Herstein, T.J. Cieslak, C. Achutan, J.J. Lowe);; University of Nebraska Medical Center College of Nursing, Omaha (E.L. Beam);; University of Nebraska Medical Center College of Medicine, Omaha (J.V. Lawler, J.L. Santarpia);; National Strategic Research Institute, Omaha (J.V. Lawler, J.L. Santarpia, G.W. Carter); University of Nebraska–Lincoln Charles W. Durham School of Architectural Engineering and Construction, Lincoln, Nebraska, USA (T.L. Stentz, K.R. Kopocis-Herstein)

**Keywords:** Ebolavirus, communicable diseases, patient isolation, transportation of patients, viruses, aeromedical transport, Ebola, Ebola virus disease, EVD

## Abstract

Circumstances exist that call for the aeromedical evacuation high-level containment transport (AE-HLCT) of patients with highly hazardous communicable diseases. A small number of organizations maintain AE-HLCT capabilities, and little is publicly available regarding the practices. The time is ripe for the development of standards and consensus guidelines involving AE-HLCT.

During a highly hazardous communicable diseases (HHCD) outbreak, most patients are likely to be treated within the affected region. However, circumstances exist for which aeromedical evacuation high-level containment transport (AE-HLCT) of patients with HHCDs is necessary (e.g., political considerations, resource limitations, armed conflict). Although AE-HLCT has occurred since the 1970s, the 2013–2016 Ebola virus disease (EVD) outbreak in West Africa brought this practice to the forefront of public consciousness (*1*). During that outbreak, at least 33 patients with confirmed or suspected EVD were transported to EVD treatment facilities in the United States and Europe that were capable of high-level isolation. Asymptomatic persons with high-risk exposures (e.g., needle sticks, blood or body fluid splashes) have also been transported for quarantine. In 2016, two patients with Lassa fever were transported by AE-HLCT from Togo, 1 each to the United States and Germany (*2*). Despite the introduction of an EVD vaccine, the Democratic Republic of the Congo is experiencing the second largest EVD outbreak in history; in December 2018, a US citizen exposed to EVD was transported to the University of Nebraska Medical Center for observation before being discharged in January 2019 (*3*). Clearly, AE-HLCT is an ongoing need.

The death rate from certain HHCDs can exceed 70%, as was the case with EVD in parts of West Africa (*4*). Thus, streamlined policies and procedures for efficient, timely AE-HLCT can decrease transmission risk and optimize patient management in flight. Because of the significant risks to crews and receiving communities, HHCD patients must be managed by highly trained teams and organizations, especially considering the uncontrolled environment of AE-HLCT missions and the potential for the rapid deterioration of patient condition. Historically, AE-HLCTs for HHCDs have been conducted by a limited number of military organizations or private corporations contracted by national governments or relief organizations to protect the well-being of their citizens or their volunteer personnel. Providers of AE-HLCT maintain teams of highly trained staff and regularly test, validate, and exercise their systems and procedures to ensure mission readiness. Moreover, these experienced organizations have recently developed systems with the capability of transporting multiple patients of varying levels of acuity during the same operation, expanding the capacity of AE-HLCT missions that historically had been limited to a single patient (*5**,**6*).

Isolation of HHCD patients during aeromedical evacuation is a complex process with numerous requirements related to the preflight, in-flight, and postflight environments, involving highly knowledgeable and trained persons from a variety of professions, as well as specialized equipment and validated infection control processes (Table) (*7*). The safe and successful use of these AE-HLCT systems requires coordination and approval at all levels of government, as well as between governments, given that most AE-HLCT missions will cross international borders. Despite this complexity, no generally accepted standards exist outside the few organizations that have conducted them. Moreover, the processes, operations, and capabilities used, as well as the lessons learned from postmission evaluations, are not found in the peer-reviewed literature. Only a few case studies have been publicly disseminated because of security concerns, a desire to maintain proprietary information, or simply because of the niche field and limited audience. The literature is sparser still regarding the methods associated with the transportation of asymptomatic persons with high-risk exposures. Although we are aware that cooperation between these organizations often occurs, the lack of literature disseminated to the larger academic and practice community remains a problem. Without knowledge of the science that established these procedures or a broad continual review of such science, an inherent barrier persists for current or future researchers or practitioners attempting to build on ongoing research and experiences.

**Table:** Processes to be considered during an aeromedical evacuation high-level containment transport

Environment, process to consider
Preflight
Types of diseases
Decision to aeromedically evacuate
Training/drills
Regulations and legal limitation
Communication plan
Layout/space assessment
Other preparations
In-flight
Personnel
Personal protective equipment
Type of isolation units
Procedures/capabilities inflight
Liquid and solid waste handling
Death in flight
Other contingency procedures
Postflight
Decontamination
Equipment reuse
Waste disposal
Personnel monitoring

Finally, the limited amount of information about the processes, procedures, and equipment available from a small number of aeromedical organizations impedes scalability should the need arise. Most of the organizations that have historically conducted AE-HLCT missions often have limited capacity, personnel, or systems to conduct multiple missions, with most only able to conduct 1 or 2 AE-HLCT missions simultaneously. The lack of nonorganizational specific standards and specialization diminishes the ability to transfer such capabilities to other organizations that might have the desire and personnel to assist or to nations that currently lack such capabilities but might have a current or future need for such capabilities. A critical evaluation of the literature would enable the dissemination of lessons learned, thereby enhancing best practices and driving the field forward, ultimately leading to safer outcomes for patients, caregivers, and receiving communities. Because much of this information does not exist within peer-reviewed literature, much would be gained through a conference on the subject that evaluates various procedures and establishes consensus recommendations for best practices, including creation of a verified information exchange mechanism. The time is ripe for the development of standards and consensus guidelines involving AE-HLCT.
